# Aryliodonium Ylides as Novel and Efficient Additives for Radical Chemistry: Example in Camphorquinone (CQ)/Amine Based Photoinitiating Systems

**DOI:** 10.3390/molecules24162913

**Published:** 2019-08-11

**Authors:** Julie Kirschner, Julien Paillard, Mariem Bouzrati-Zerelli, Jean-Michel Becht, Joachim E. Klee, Saloua Chelli, Sami Lakhdar, Jacques Lalevée

**Affiliations:** 1 Institut de Science des Matériaux de Mulhouse IS2M, UMR CNRS 7361, UHA, 15 rue Jean Starcky, 68057 Mulhouse CEDEX, France; 2 Dentsply Sirona, De-Trey-Straβe 1, 78462 Konstanz, Germany; 3 Normandie Univ., LCMT, ENSICAEN, UNICAEN, CNRS, 6, Boulevard Maréchal Juin, 14000 Caen, France

**Keywords:** photoinitiator, iodonium salt, radical initiation, photopolymerization

## Abstract

Diaryliodonium salts are well-established compounds in free radical chemistry and are already used as photoinitiators (free radical or cationic polymerization), but the presence of counter anions is a strong drawback. Indeed, a counter anion is always required (e.g., SbF_6_^−^) leading to potential toxicity issues or release of HF. In the present paper, counter anion-free and fluoride-free aryliodonium salts are proposed, that is, aryliodonium ylides (AY) are studied here as new and efficient additives for radical chemistry and an example is provided for the camphorquinone (CQ)/amine based photoinitiating systems (PISs) for the polymerization of thick (1.4 mm) and thin (20–13 µm) methacrylates under air and blue light irradiation. The newly proposed PISs, for example, CQ/amine/AY, presented excellent polymerization performances and good bleaching properties were obtained after polymerization. Real-time Fourier transform infrared spectroscopy (RT-FTIR) was used to monitor the photopolymerization profiles. The chemical mechanisms involved were investigated using electron spin resonance (ESR).

## 1. Introduction

Photoinitiated polymerization has been widely used in the past decades and has found many industrial applications, such as 3D printing, coatings, inks, adhesives, and so on. Two types of polymerization can be distinguished depending on the nature of the photoinitiator used; free radical photopolymerization and cationic polymerization [1,2]. The latter presents numerous advantages compared to the former, such as insensitivity to oxygen, low shrinkage during curing, dark polymerization after the removal of the light source, and so on [3] Two families of cationic photoinitiators are generally used: iodonium and sulfonium salts [1,4,5]. As iodonium salts present better solubility in non-polar monomers than the corresponding sulfonium salts, they are the most widespread cationic photoinitiators [6]. Iodonium salts are also usually very efficient, but absorb at shorter wavelengths. Diphenyliodonium salts associated with BF_4_^−^, PF_6_^−^, AsF_6_^−^, SbF_6_^−^ as counter anions are the simplest and the most frequently used [1,6]. The cationic part of the diphenyliodonium salt is responsible for light absorption whereas the nature of the counter anion governs the strength of the acid formed during photolysis [1]. Nevertheless, the diaryliodonium photoinitiators used currently all exhibit absorption properties in the UV absorption range between 220 and 280 nm while there are no light sources efficient enough operating in this spectral range [1,6,7,8,9]. To overcome their poor absorption properties in the visible range, diaryliodonium salts can be used in combination with a photosensitizer (PS) and/or a visible free radical photoinitiator [10]. As proposed by Crivello, diaryliodonium salts can be sensitized by the PS through an electron or energy transfer process [11]. After excitation of the PS and reduction of the diaryliodonium salt, aryl radicals are generated through the cleavage of the C–I bond [11,12,13,14,15].

On the other hand, two types of photoinitiator (PI) are used in free radical photopolymerization: Type I and Type II PI [1]. Due to its good visible absorption properties, camphorquinone is the most common Type II PI used for the free radical photopolymerization of (meth-)acrylates under blue light irradiation. To be efficient, camphorquinone has to be used in combination with a hydrogen donor, for example, an amine [16,17,18]. The performances of the camphorquinone (CQ)/amine system can also be improved in the presence of well-chosen additives, such as diaryliodonium salts, as they generate initiating aryl radicals upon light irradiation [1]. Diaryliodonium hexafluorophosphates (e.g., diphenyliodonium hexafluorophosphate, Speedcure 938, and so on) are frequently used diaryliodonium salts, due to their commercial availability [19,20]. Nevertheless, in certain conditions, the hexafluorophosphate counter anion may cause some issues due to the release of HF [21,22].

The idea of this paper is to develop new counter anion-free and fluoride-free aryliodonium salts to avoid the formation of side products, for example, formation of HF and to enhance their solubility in monomers. In this paper, we describe two aryliodonium ylides (S5 and S6) as new efficient aryliodonium salts and additives for CQ/amine based systems for the free radical polymerization of methacrylates under visible light irradiation. Aryliodonium ylides (AY) were studied for the replacement of traditionally used diaryliodonium salts (diphenyliodonium salt, Speedcure 938, and so on) in the CQ/amine system. The performances of the newly proposed CQ/amine/AY systems for the free radical photopolymerization of thick and thin samples of methacrylates were evaluated using real-time FTIR experiments. Electron spin resonance (ESR) spectroscopy was used to identify the radicals generated upon light irradiation. A chemical mechanism between CQ and aryliodonium ylide is proposed.

## 2. Materials and Methods

### 2.1. Materials

**Scheme 1:**
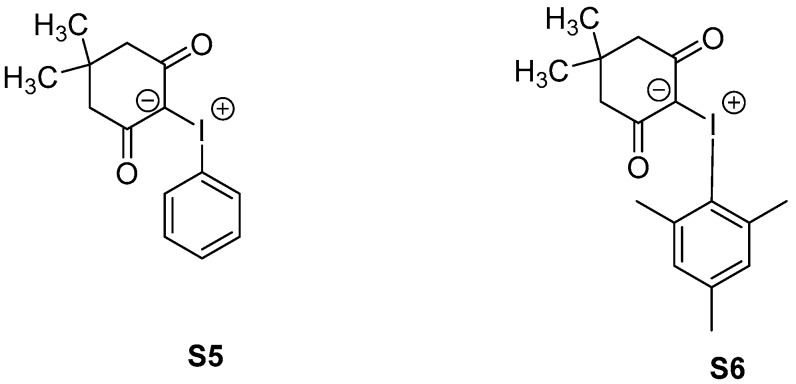
Structures of the aryliodonium ylides (AY) studied: S5 and S6.

**Scheme 2:**
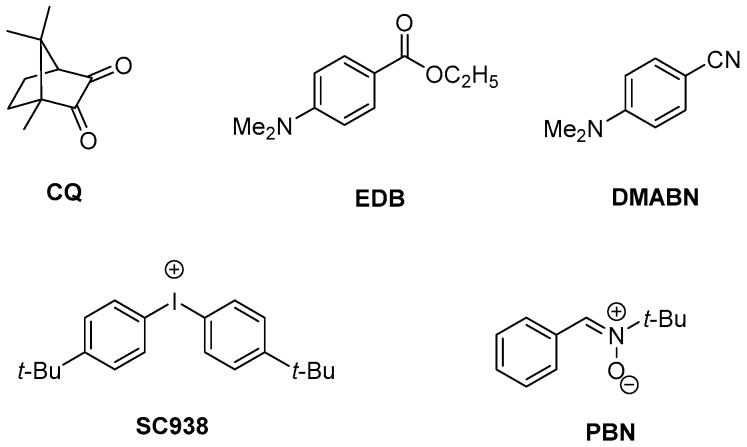
Structures of the photoinitiator (camphorquinone; CQ) and additives/co-initiators used.

S5 and S6 (Scheme 1) were prepared according to a synthetic procedure already described in the literature [23]. Camphorquinone was obtained from Sigma Aldrich (St. Louis, MO, USA) and used as a representative Type II PI (Scheme 2). Ethyl-4-(dimethylamino)benzoate (EDB) and 4-(dimethylamino)benzonitrile (DMABN) were used as additives in multicomponent systems and obtained from Sigma Aldrich or TCI Chemicals (Scheme 2). Speedcure938 (SC938) was obtained from Lambson Ltd. (Wetherby, UK, Scheme 2). Phenyl-*N*-*tert*-butylnitrone (PBN, Scheme 2) was used as spin trapping agent in the ESR experiments.

**Scheme 3:**
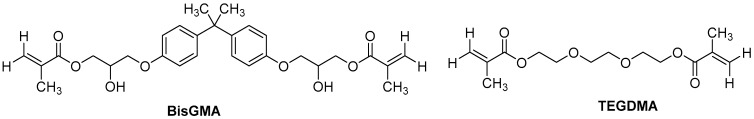
Structures of the monomers used.

Spectrum^®^ TPH^®^3 resin was received from Dentsply Sirona (Konstanz, Germany) and used as a representative matrix of dental materials, consisting of a mixture of modified BisGMA, TEGDMA (Scheme 3) and other methacrylate monomers. PrimeBond Active^®^ was received without photoinitiator from Dentsply Sirona and used as a representative formulation for the preparation of dental adhesives.

### 2.2. Synthesis of Aryliodonium Ylides S5 and S6

Synthesis of S5: iodobenzene diacetate (0.20 g, 0.62 mmol, 1 equiv) was dissolved in ethanol 96 % (1 M, 0.62 mL) under heating and added quickly to a dimedone (0.087 g, 0.62 mmol, 1 equiv) dissolved in a saturated aqueous solution of sodium carbonate (0.3 M, 1.90 mL). The resulting mixture was stirred for 30 min at RT. Cold water (2 mL) was then added and the aqueous phase was extracted 3 times with DCM (3 × 10 mL). The organic phase was dried with anhydrous MgSO_4_ and concentrated under reduced pressure to give S5 (0.12 g, 0.35 mmol, 57 % isolated yield) as a white solid. ^1^H NMR (CDCl_3_, 300 MHz) δ (ppm): 7.77 (d, *J* = 6 Hz, 2H), 7.47 (m, 1H), 7.31 (m, 2H), 2.45 (s, 4H), 1.02 (s, 6H); ^13^C NMR (CDCl_3_, 50 MHz) δ (ppm): 188.4, 133.9, 131.5, 112.0, 94.6, 50.8, 32.1, 28.2; HRMS (ESI MS) *m*/*z*: calculated: 343.0190 found: 343.0188 ((M + H)^+^ detected).

Synthesis of S6: dimedone (0.19 g, 1.37 mmol, 1 equiv) was dissolved in a saturated solution of sodium carbonate (0.3 M, 1.70 mL). Iodomesitylene diacetate (0.50 g, 1.37 mmol, 1 equiv) was dissolved in ethanol 96 % (1 M, 1.37 mL) under heating. The solution of iodomesitylene was quickly added to the solution containing the conjugated base of dimedone. The resulting mixture was stirred for 30 min at RT. Cold water (2 mL) was then added and the aqueous phase was extracted 3 times with DCM (3 × 10 mL). The organic phase was dried with anhydrous MgSO_4_ and concentrated in vacuo to give S6 (0.34 g, 0.88 mmol, 64 % isolated yield) as a light yellow powder. ^1^H NMR (CDCl_3_, 300 MHz) δ (ppm): 6.96 (s, 2H), 2.72 (s, 6H), 2.42 (s, 4H), 2.28 (s, 3H), 1.02 (s, 6H); ^13^C NMR (CDCl_3_, 50 MHz) δ (ppm): 187.9, 142.6, 129.5, 93.4, 50.9, 32.0, 28.1, 27.3; HRMS (ESI MS) *m*/*z*: calculated: 385.0659 found: 385.0660 ((M + H)^+^ detected).

### 2.3. Irradiation Sources

**Figure 1:**
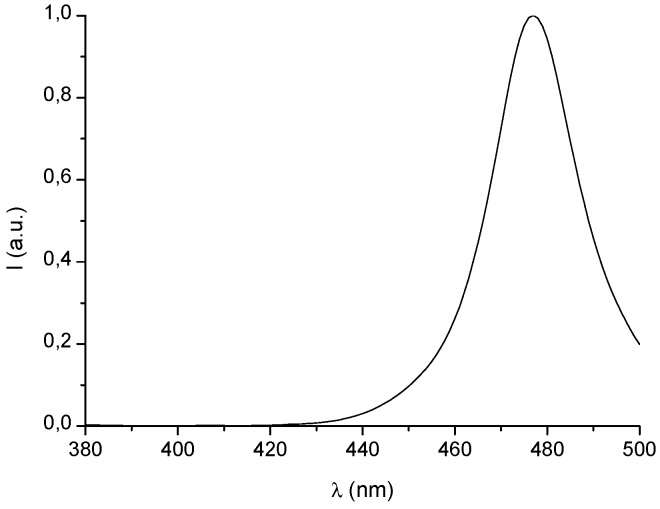
The emission spectrum of a blue LED centered at 477 nm (SmartLite Focus from Dentsply Sirona Germany).

A blue LED@477nm representative of dental materials usage (SmartLite Focus from Dentsply Sirona ~300 mW·cm^−2^ in the selected conditions; see emission spectrum in Figure 1) and an LED centered at 455 nm (M455L3-ThorLabs; ~80 mW·cm^−2^) were used for the irradiation of the photocurable samples.

### 2.4. Absorption Experiments

The UV-visible spectra were recorded using a JASCO V-730 UV/vis spectrophotometer (JASCO, Japan).

### 2.5. Photopolymerization Experiments

The photosensitive formulations were deposited on a polypropylene film and the sample thickness was controlled using a mold (1.4 mm thick samples). The samples were polymerized under air and irradiation with the LED source. The evolution of the methacrylate functions was continuously followed by real time FTIR spectroscopy (JASCO FTIR 6600, JASCO, Japan) at about 6165 cm^−1^ for thick (1.4 mm) methacrylate samples and at 1220 cm^−1^ for thin (13–20 µm) ones.

### 2.6. ESR Spin Trapping Experiments:

ESR experiments were carried out using a Bruker EMX-plus spectrometer (X-band, Bruker Company, Rheinstetten, Germany). The radicals were generated at room temperature upon the blue LED exposure (SmartLite Focus from Dentsply Sirona) under N_2_. The radicals were trapped by phenyl-*N*-*tert*-butylnitrone (PBN, Scheme 2) according to a procedure already described [24]. The ESR spectra simulations were carried out using WINSIM software.

## 3. Results and Discussion

### 3.1. Photochemical Mechanism

**Figure 2:**
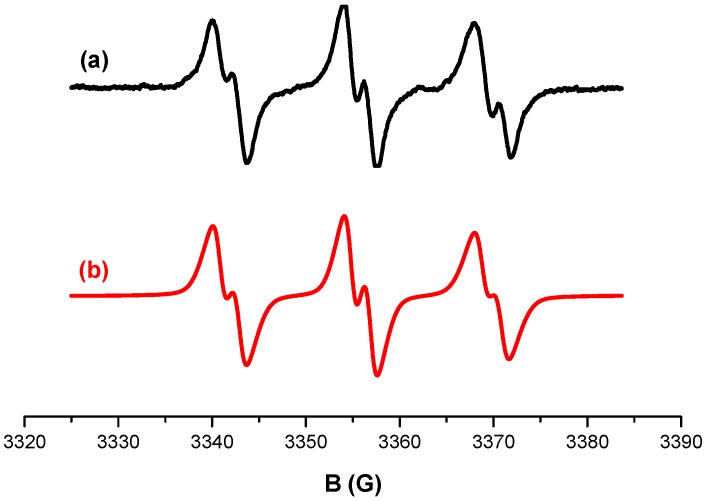
Electron spin resonance (ESR) spectra of the radicals generated in CQ/S5 (0.05 M/0.01 M) and trapped by phenyl-*N*-*tert*-butylnitrone (PBN) (0.01M) in *tert*-butylbenzene upon exposure to a LED@477 nm (SmartLite Focus): N_2_ saturated medium Ph^•^/PBN hyperfine coupling constants: a_N_ = 13.96 G, a_H_ = 2.06 G; (**a**) experimental and (**b**) simulated spectra.

In the presence of a spin trap agent (PBN) for the photolysis of CQ/S5 under N_2_ upon exposure to the blue LED centered at 477 nm, the Ph^•^/PBN radical adducts were clearly observed (i.e., characterized by hyperfine coupling constants (hcfs) a_N_ = 14.0 G, a_H_ = 2.1 G in agreement with hfcs known in the literature [25] (Figure 2)). Therefore, ESR spin trapping experiments showed the formation of phenyl radicals generated from the CQ/S5 interaction upon blue light. This result clearly highlights the cleavage of the C–I central bond of S5 and the possible release of benzene by the decomposition of S5 (through hydrogen abstraction process with the generated phenyl radical in S5). As benzene release must be clearly avoided in industrial applications for safety reasons, a derivative of S5 was prepared where the phenyl group was replaced by a 2,4,6-trimethylphenyl (mesityl) group: S6.

A general chemical mechanism for aryliodonium ylides can be proposed in agreement with the result obtained for the ESR spin trapping experiment for CQ/S5. Aryl radicals were generated according to reaction 1 (r1) through an electron transfer between camphorquinone and the aryliodonium ylide (Ar–I–R).

*CQ + Ar–I–R → CQ^●−^ + Ar^●^ + R = I^+^ (r1)

**Figure 3:**
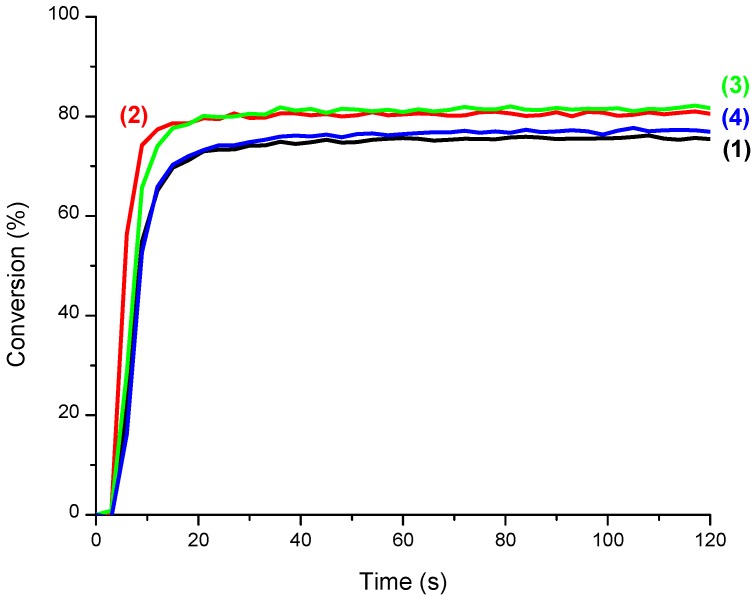
Photopolymerization profiles (methacrylate function conversion vs. irradiation time) for Spectrum^®^ TPH^®^3 resin (under air; 1.4 mm thick films) upon exposure to the LED@477 nm (I_0_ = 300 mW·cm^−2^) using different photoinitiating systems. (1) CQ/ethyl-4-(dimethylamino)benzoate (EDB) (0.2/0.5 % *w*/*w*); (2) CQ/EDB/SC938 (0.2/0.5/1 % *w*/*w*); (3) CQ/EDB/S5 (0.2/0.5/1 % *w*/*w*); (4) CQ/EDB/S6 (0.2/0.5/1 % *w*/*w*). The irradiation starts for t = 5 s.

**Figure 4:**
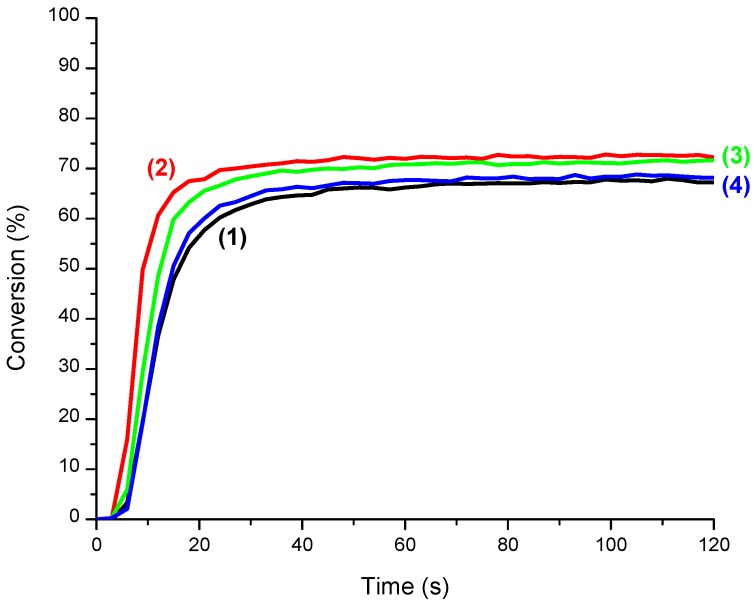
Photopolymerization profiles (methacrylate function conversion vs. irradiation time) for a Spectrum^®^ TPH^®^3 resin blend (under air; 1.4 mm thick films) upon exposure to the LED@455 nm (I_0_ = 80 mW·cm^−2^) using different photoinitiating systems. (1) CQ/EDB (0.2/0.5 % *w*/*w*); (2) CQ/EDB/SC938 (0.2/0.5/1 % *w*/*w*); (3) CQ/EDB/S5 (0.2/0.5/1 % *w*/*w*); (4) CQ/EDB/S6 (0.2/0.5/1 % *w*/*w*). The irradiation starts for t = 5 s.

Aryl radicals could initiate the free radical polymerization of (meth)acrylate monomers in agreement with the good initiating ability of CQ/EDB/S5 vs. CQ/EDB found in polymerization experiments (see below, Figure 3 and Figure 4).

In the presence of amine (EDB, DMABN), additional reactions were expected [1] r2–r4 leading to additional initiating radicals.

*CQ + Me_2_NAr → CQ–H^•^ + Me(CH_2_^•^)NAr (r2)

CQ–H^•^ + Ar–I–R → → CQ-H^−^ + Ar^•^ (r3)

CQ–H^•^ + Ar–I–R → → CQ + H^+^ + Ar^•^ (r4)

### 3.2. Absorption Properties 

**Figure 5:**
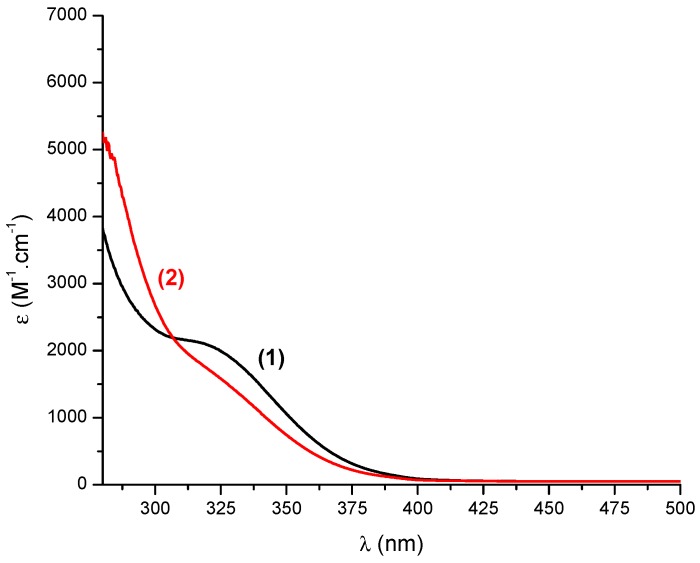
UV-visible spectra of (1) S5 and (2) S6 in acetonitrile.

The absorption properties of diaryliodonium salts are well known in the literature, for example, for diphenyliodonium the maximum absorption wavelength is located at ~230 nm [1]. The UV-visible spectra of S5 and S6 present a broad band in the 300–400 nm region (Figure 5). Contrary to the well-known diphenyliodonium, S5 and S6 present shifted absorption spectra to the near UV region with maximum absorption wavelengths located at ~330 nm. Therefore, compared to diphenyliodonium salt, the aryliodonium ylides S5 and S6 lead to a shift of the absorption spectra to longer wavelengths. Remarkably, the newly proposed aryliodonium ylides present rather good absorption properties at 365 nm (ε: 532 M^−1^.cm^−1^ for S5 and 367 M^−1^.cm^−1^ for S6) matching well with the emission spectra of common mercury lamps used in the coatings industry, and could therefore be used as photoinitiators in this application. For industrial fields using blue light irradiation, for example, dentistry, S5 and S6 must be sensitized by camphorquinone to form initiating radicals under visible light irradiation. Accordingly, no radicals were observed in ESR experiments upon blue light without camphorquinone.

### 3.3. CQ/EDB/S5 and CQ/EDB/S6 Initiating Ability

The polymerization profiles of thick films (1.4 mm) of Spectrum^®^ TPH^®^3 resin upon exposure to the blue LED at 477 nm (SmartLite Focus) using different photoinitiating systems are presented in Figure 3. Under air, using a 300 mW·cm^−2^ light intensity, the systems CQ/EDB/S5 and CQ/EDB/S6 exhibited very good polymerization ability in thick samples. High polymerization rates Rp and high final methacrylate conversions (FCs) up to 80 % were obtained for the systems CQ/EDB/S5 and CQ/EDB/S6 (curves 3–4 Figure 3). Remarkably, similar or better performances were obtained for the two PISs investigated, compared to the reference systems CQ/EDB and CQ/EDB/SC938 (curves 1–2 Figure 3).

Similar results were obtained using an LED centered at 455 nm for the polymerization of thick (1.4 mm) samples of Spectrum^®^ TPH^®^3 resin under air. The polymerization profiles of thick films (1.4 mm) of Spectrum^®^ TPH^®^3 resin upon exposure to the LED at 455 nm using different photoinitiating systems are presented in Figure 4. Remarkably, the CQ/EDB/S5 and CQ/EDB/S6 systems lead to higher final conversions and polymerization rates than the reference system CQ/EDB.

**Figure 6:**
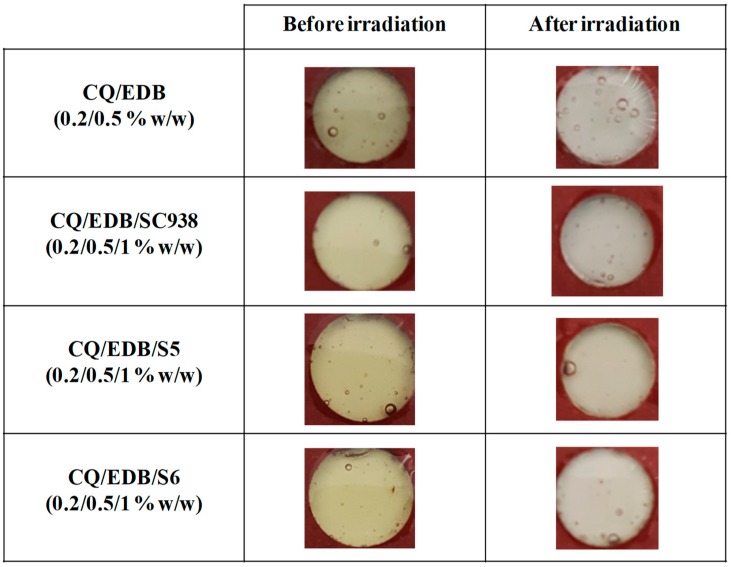
Photos of the samples before and after polymerization (under air; thickness = 1.4 mm; SmartLite Focus (300 mW·cm^−2^); 115 s irradiation): (1) CQ/EDB (0.2/0.5 % *w*/w); (2) CQ/EDB/SC938 (0.2/0.5/1 % *w*/*w*); (3) CQ/EDB/S5 (0.2/0.5/1 % *w*/*w*); (4) CQ/EDB/S6 (0.2/0.5/1 % *w*/*w*) in Spectrum^®^ TPH^®^3.

Remarkably, good bleaching properties were obtained with the systems CQ/EDB/S5 and CQ/EDB/S6 compared to the references CQ/EDB and CQ/EDB/SC938 after 115s of irradiation with the blue LED centered at 477 nm. Similar or better bleaching properties are obtained with the newly developed PISs compared to those achieved with the reference PISs (Figure 6).

### 3.4. CQ/EDB/S5 and CQ/EDB/S6 for the Polymerization of Thin Samples of Methacrylates and Adhesives under Air

**Figure 7:**
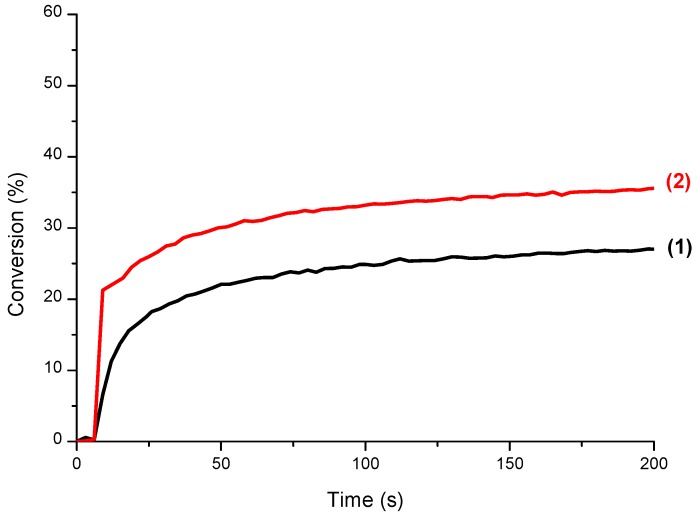
Photopolymerization profiles (methacrylate function conversion vs. irradiation time) for a BisGMA/TEGDMA (70/30 % *w*/*w*) resin blend (under air; 20 µm thin films) upon exposure to the LED@477 nm (I_0_ = 300 mW·cm^−2^) using different photoinitiating systems. (1) CQ/EDB (1/1 % *w*/*w*); (2) CQ/EDB/S5 (1/1/1 % *w*/*w*). The irradiation starts for t = 5 s.

The newly proposed PISs CQ/EDB/S5 and CQ/EDB/S6 showed excellent polymerization performances for the polymerization of thick (1.4 mm) samples of methacrylates under air upon exposure to a blue LED centered at 477 nm and an LED centered at 455 nm. The performances of these systems were also investigated for the preparation of thin (20 µm) samples of methacrylates under exposure to the LED centered at 477 nm (Figure 7). Remarkably, the system CQ/EDB/S5 presented an enhanced polymerization rate and final methacrylate function conversion (~35 % vs. 25 %) compared to the reference system CQ/EDB for polymerization under air. The lower conversion in Figure 7 compared to Figure 3 and Figure 4 was ascribed to the oxygen inhibition that is stronger for thin samples compared to thick ones.

**Figure 8:**
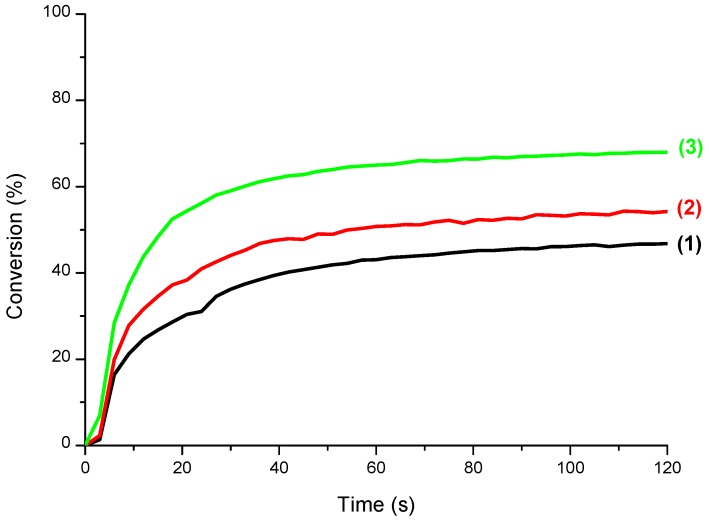
Photopolymerization profiles (methacrylate function conversion vs. irradiation time) for Prime&Bond Active^®^ resin (under air; 13 µm thin films) upon exposure to the LED@477 nm (I_0_ = 300 mW·cm^−2^) using different photoinitiating systems. (1) CQ/4-(dimethylamino)benzonitrile (DMABN) (1.5/0.6 % *w*/*w*); (2) CQ/DMABN/S5 (1.5/0.6/0.75 % *w*/*w*); (3) CQ/DMABN/S6 (1.5/0.6/0.75 % *w*/*w*). The irradiation starts for t = 5 s.

The newly proposed systems were also investigated for the preparation of very thin (~13 µm) samples of adhesives by polymerization under air under exposure to the blue dental LED. The polymerization profiles of thin adhesive films (~13 µm) of Prime&Bond Active^®^ formulation upon exposure to the blue LED at 477 nm (SmartLite Focus) using different photoinitiating systems are depicted in Figure 8. Remarkably, better polymerization performances (i.e., higher final methacrylate function conversions and higher polymerization rates) were achieved for the systems CQ/DMABN/S5 and CQ/DMABN/S6 compared to the reference system CQ/DMABN. The new systems proposed presented excellent performances for the polymerization in difficult conditions, for example, for very thin samples and under air.

## 4. Conclusions

In the present paper, aryliodonium ylides (AY) were reported as new high performance iodonium salts and efficient additives for CQ/amine based systems for the polymerization of methacrylates under blue light irradiation. Enhanced polymerization performances were achieved for the system CQ/amine/AY compared to the reference system CQ/amine. Good bleaching properties of the final polymers were also obtained after polymerization. Interestingly, the newly developed CQ/amine/AY systems presented excellent initiating ability in strongly oxygen-inhibited conditions (thin samples, under air), for example, for the preparation of adhesives. Aryliodonium ylides therefore represent a new class of iodonium salts for photoinitiated polymerization and an interesting alternative to the well-known diaryliodonium salts. Remarkably, as the counter anion is located on the iodonium moiety, enhanced solubility in monomers is expected, as well as reduced formation of side products, compared to the classical diaryliodonium salts, especially diaryliodonium hexafluorophosphate.
